# Patient-mediated scale-up of guideline implementation in primary-care: an open-label trial

**DOI:** 10.1186/1748-5908-10-S1-A7

**Published:** 2015-08-14

**Authors:** Peter Selby, Laurie Zawertailo, Rosa Dragonetti, Sarwar Hussain, Sabrina Voci

**Affiliations:** Centre for Addiction and Mental Health, Toronto, Ontario M5T 1P7 Canada

## Objective

To advance the knowledge on how to amplify the impact of effective clinical interventions to the population level, we tested an approach that took the traditional model of care delivery, which relied on busy physician practices to initiate treatment and transformed it into a patient-driven model, enabled by web technology and strategic labor distribution among patient, physician and pharmacist. Varenicline and bupropion are effective pharmacotherapies for smoking cessation, but many clinicians do not proactively discuss these options with their smoker patients. Using cost-free medication as an incentive, our objective was to demonstrate the feasibility of enrolling smokers via the internet in a protocol to engage their family physician in a discussion of smoking cessation treatment with pharmacotherapy.

## Method

Participants visited the study website, provided consent and completed an on-line assessment. Eligible participants received a personalized script to take to their physician, who had the option of prescribing bupropion or varenicline for 12 weeks. Signed scripts were faxed by the physician's office to a central pharmacy that couriered the medication to the patient. Pharmacist provided one session of brief telephone counseling.

## Results

In 2 months, 893 participants enrolled, of whom 524 (59%) visited 419 different physicians to have the script signed and faxed (265 varenicline, 259 bupropion). All medication packages were delivered, with only 1.0% (n = 5) requiring address clarification or reshipment. At 6-month follow-up, the 7-day point prevalence of abstinence was 30.3% for varenicline vs. 24.3% for bupropion (p >0.1; OR (95%CI): 1.4 (0.8-2.2)).

## Conclusion

These quit rates are comparable to those seen in randomized clinical trials. The e-patient movement with the world-wide-web as its forum has patients acting as drivers of their healthcare. This emerging characteristic of population behavior provides a new opportunity for rapid scaling-up of implementation of evidence to the state-or national-level that is otherwise extended to limited practice settings. This study was funded by the Ontario Ministry of Health and Long-Term Care.

